# Novel indole diketopiperazine stereoisomers from a marine-derived fungus *Aspergillus* sp

**DOI:** 10.1080/21501203.2022.2069173

**Published:** 2022-04-29

**Authors:** Xinyang Li, Jinzhong Xu, Pinmei Wang, Wanjing Ding

**Affiliations:** Institute of Marine Biology and Pharmacology, Ocean College, Zhejiang University, Zhoushan, China

**Keywords:** Indole diketopiperazine, dimer, stereoisomers, gut fungus

## Abstract

Four dimeric diketopiperazine stereoisomers (**1–4**) including two new ones (**1–2**) had been isolated from the culture broth of one marine-derived fungus *Aspergillus* sp. Z3, which was found in the gut of a marine isopod *Ligia exotica*. The planner structures and absolute configurations of the new compounds were determined by combination of NMR, HRESIMS, electronic circular dichroism calculation, Marfey’s method as well as single-crystal X-ray diffraction. Their cytotoxicity against the prostate cancer PC3 cell line was assayed by the MTT method.

## 1. Introduction

**Figure 1.**
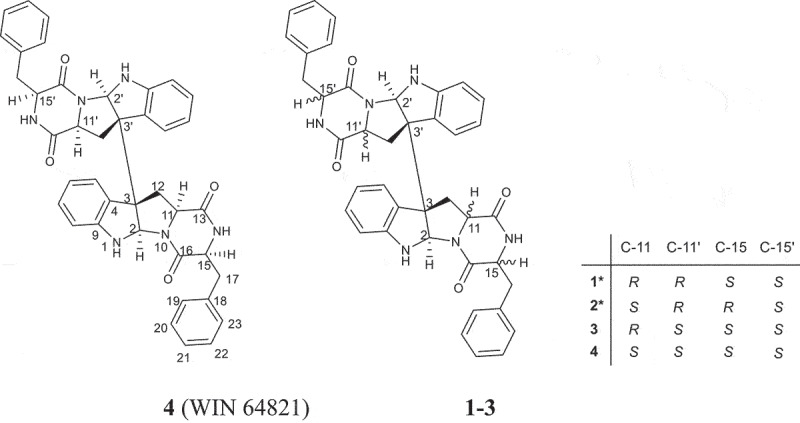
Structures of compounds **1–4.**

Indole diketopiperazines, which were formed by the condensation of tryptophan with other different amino acids, are an important family of secondary metabolites commonly produced by fungi. Most of these alkaloids are characterised by diverse structural scaffolds and ring systems derived from dimerisation by two indole units and conjugation with another building block as cyclic dipeptides (Li et al. 2009; Ma et al. 2016; Klas et al. 2018). This class of alkaloids possess diverse biological activities such as antimicrobial (Zheng et al. 2007; Jia et al. 2019; Yang et al. 2021), anticancer (Wang et al. 2015; Zhang et al. 2019), immunomodulatory (Fujimoto et al. 2004), anti-inflammatory (Barrow et al. 1993; Wen et al. 2018; Yang et al. 2021), antioxidant (Sun et al. 2018) and antiviral (Wibowo et al. 2021; Nishiuchi et al. 2022), which attracted much attention. Our ongoing research on marine fungal metabolites revealed one diketopiperazine dimer WIN 64821 (**4**), a competitive antagonist to substance P (Barrow et al. 1993) and an antagonist of cholecystokinin type-B receptor (Sedlock et al. 1994), with a series of its stereoisomers (**1–3**) from the marine-derived fungus *Aspergillus* sp. Z3 (Figure 1). Compounds **1** and **2** are new ones. Herein, the purification, structure elucidation, and biological activities of the four compounds are described.

## 2. Materials and methods

### 2.1. General experimental procedures

Optical rotation was measured on an Autopol I polarimeter (Rudolph Research Analytical, Hackettstown, NJ, USA). The ultraviolet (UV) absorption spectra were measured in MeOH on a METASH UV-8000 spectrophotometer (Shanghai METASH Instruments Co. Ltd., Shanghai, China). The electronic circular dichroism (ECD) was measured on a JASCO J-815 spectropolarimeter (JASCO Co. Tokyo, Japan). The infrared (IR) spectra were recorded using a Bruker Vector 32 spectrometer (Brucker, Karlsruhe, Germany). NMR spectra were recorded in CDCl_3_ (ALDRICH, St. Louis, MO, USA) or MeOH-d_4_ (ALDRICH, St. Louis, MO, USA) with tetramethylsilane (TMS) as an internal standard, using a Bruker AV III 500 MHz NMR spectrometer. HR-ESIMS data were obtained on an Agilent 6224 TOF LC/MS. Single crystal X-ray crystallography was determined on an Xcalibur Atlas Gemini Ultra diffractometer (Agilent Technologies). Column chromatography (CC) was performed with silica (100–200 and 200–300 mesh, Qingdao Haiyang Chemical Co., Ltd., Qingdao, China).

### 2.2. Fungal material and fermentation

The fungal colony (Z3) was isolated from marine isopod *Ligia exotica* which was collected in seaside of Dinghai in Zhoushan, Zhejiang Province of China, in December 2011. The fungal Z3 was determined as *Aspergillus* sp. by ITS analysis.

The strain (Z3) was static cultured at 28°C for 30 days in 500 mL Erlenmeyer-flasks (200 × 250 mL, a total of 50 L) each containing 250 mL of 2216E liquid media (QingDao Hopebio-Technology Co., Ltd., Qingdao, China).

The culture broth (50 L) was filtered and extracted with equivoluminal EtOAc for 3 times to obtain 5 g metabolites extract, which was fractioned by silica gel column chromatography (CC) eluted in gradient from cyclohexane- EtOAc (20:1–10:1) to CH_2_Cl_2_- MeOH(100:1–5:1). Ten fractions (Frs. 1–10) were collected based on TLC analysis. Fr. 8 (500 mg) was purified by preparative ODS-HPLC (Agilent Persuit XRs C18, 10 μm, 21.2 × 250 mm, 10 mL/min, 60% MeOH in H_2_O) to obtain compound **3** (44.2 mg), **4** (44.2 mg) and **1** (6.2 mg). Fr. 9 (230 mg) was purified by semi-preparative ODS-HPLC (COSMOSIL PACKED COLUMN, 5C18-MS-II column, 10ID×250 mm, 4 mL/min, 53% MeOH in H_2_O) to obtain compound **2** (7.2 mg).

#### 2.2.1. Physicochemical and spectral data

Compound **1**:white solid; [*α*] ^25^_D_ +478.0 (*c* 1.1, CHCl_3_); UV (MeOH) *λ*_max_ (log*ε*): 210 (3.9), 241 (2.3), 300 (1.4) nm; IR (film) *ν*_max_: 3380, 2924, 1667, 1408, 1340, 1255 cm^−1; 1^H and ^13^C NMR data see Tables 1 and 2; (+)-HRESIMS [M + H]^+^ *m/z* 665.2868 (calcd. 665.2871 for C_40_H_37_N_6_O_4_).

**Table 1.** ^1^H NMR data for compounds **1–2** (^1^H NMR at 500 MHz).

Pos.	1^b^, *δ*_H_ (*J* in Hz)	2^a, b^, *δ*_H_ (*J* in Hz)	Pos.	2^a, b^, *δ*_H_ (*J* in Hz)
1		5.70, s	1’	5.11, s
2	5.03, br s	4.83, br s	2’	4.87, br s
5	6.94, d (7.8)	7.08, m	5’	6.97, d (7.5)
6	6.72, t (7.8)	6.71, t (7.5)	6’	6.79, t (7.5)
7	7.19, td (1.3, 7.8)	7.06, m	7’	7.28, m
8	6.71, d (7.8)	6.60, d (7.5)	8’	6.76, d (7.5)
11	1.89, dd (11.4 5.7)	2.31, m	11’	2.21, m
12	2.29, t (12.3) 2.17, dd (12.3 5.9)	2.60, dd (13.5, 8.5) 2.24, m	12’	2.30, m
14		5.94, s	14’	6.02, s
15	4.13, t (4.1)	4.10, dd (8.6, 4.5)	15’	4.14, dd (8.5, 4.5)
17	3.16, dd (13.5, 3.5) 2.83, dd (13.5, 5.2)	3.19, dd (14.0, 5.0) 2.83, dd (14.0, 4.0)	17’	3.14, dd (13.5, 4.5) 2.87, dd (14.0, 4.5)
19/23	6.98, m	7.18, t (7.5)	19’/23’	6.97, d (7.7)
20/22	6.85, m	7.07, m	20’/22’	6.92, m
21	6.84, m	7.30, m	21’	6.92, m

^a^in CDCl_3_; ^b^ in CH_3_OH-*d*_4_.

^b^data of H-1- H-23 can be changed with that of H-1’~ H-23’.

**Table 2.** ^13^C NMR data for compounds **1–2** (^13^C NMR at 125 MHz).

Pos.	1^b^, *δ*_C_	2^a, b^, *δ*_C_	Pos.	2^a, b^, *δ*_C_
2	78.2	78.3	2’	80.3
3	58.0	58.1	3’	58.5
4	125.7	129.7	4’	126.4
5	124.9	126.0	5’	124.4
6	118.7	119.3	6’	119.9
7	129.6	129.2	7’	129.8
8	109.6	109.9	8’	110.0
9	150.1	148.4	9’	150.1
11	56.8	54.8	11’	56.6
12	35.0	36.7	12’	35.4
13	169.5	168.7	13’	168.9
15	57.6	58.7	15’	58.3
16	166.1	167.2	16’	165.7
17	39.9	40.2	17’	40.4
18	133.8	134.5	18’	134.0
19/23	129.6	129.1	19’/23’	129.6
20/22	128.3	129.7	20’/22’	128.7
21	126.8	128.0	21’	127.2

^a^in CDCl_3_; ^b^ in CH_3_OH-*d*_4_;

^b^data of C-1- C-23 can be changed with that of C-1’~ C-23’.

Compound **2**:white solid; [*α*] ^25^_D_ +228.4 (*c* 0.90, CHCl_3_); UV (MeOH) *λ*_max_ (log*ε*): 210 (4.3), 242 (2.6), 301 (1.7) nm; IR (film) *ν*_max_: 3378, 2925, 1670, 1437, 1316, 1097 cm^−1; 1^H and ^13^C NMR data see Tables 1 and 2; (+)-HRESIMS [M + H]^+^ *m/z* 665.2863 (calcd. 665.2871 for C_40_H_37_N_6_O_4_).

Compound **3**:white solid; [*α*] ^25^_D_ +316.8 (*c* 0.95, CHCl_3_); UV (MeOH) *λ*_max_ (log*ε*): 210 (3.9), 242 (2.2), 301 (1.1) nm; IR (film) *ν*_max_: 3385, 2923, 2851, 1667, 1435, 1254 cm^−1; 1^H and ^13^C NMR data see Table S1 and S2, Supplementary data; (+)-HRESIMS [M + H]^+^ *m/z* 665.2860 (calcd. 665.2871 for C_40_H_37_N_6_O_4_).

WIN 64821 (**4**):white solid; [*α*] ^25^_D_ +397.3 (*c* 0.95, CHCl_3_); UV (MeOH) *λ*_max_ (log*ε*): 210 (4.3), 242 (2.6), 301 (1.7) nm; IR (film) *ν*_max_: 3405, 2930, 1635, 1455, 1341, 1248 cm^−1; 1^H and ^13^C NMR data see Table S1 and S2, Supplementary data; (+)-HRESIMS [M + H]^+^ *m/z* 665.2894 (calcd. 665.2871 for C_40_H_37_N_6_O_4_).

### 2.3. Marfey’s method

Compounds **1–3** (0.5 mg) were hydrolysed in 1 mL HCl (6 M) at 110°C for 24 h, respectively. The cooled reaction mixture was evaporated to dryness and HCl was removed. The dried hydrolysate was dissolved in 120 μL ddH_2_O and then 20 μL NaHCO_3_ (1 M) and 400 μL 1% (w/v) FDAA in acetone were added to the solution of the hydrolysate. The mixture was stirred at 42°C for 2 h and the reaction was terminated by an addition of 10 μL HCl (1 M). The reaction product was evaporated to dryness and dissolved with 1 mL MeOH to give amino acid-FDAA derivatives for the HPLC analysis. Each standard amino acid (0.5 mg) of L-phenylalanine and D-phenylalanine was also converted to its FDAA derivatives. For Marfey′s analysis, each of the amino acid-FDAA derivatives (15 μL) was analysed by HPLC (SHIMADZU column: C_18_, 5 μm, 4.6 × 250 mm; flow rate: 1.0 mL/min; detection wavelength: 340 nm) with the linear gradient programme from 10% to 100% acetonitrile in ddH_2_O over 60 min. Each chromatographic peak was identified by comparing its retention time with the FDAA derivatives of the standard amino acids. The retention time of the standard derivatives was as follows: 23.2 min for FDAA, 28.8 min for L-phenylalanine and 31.1 min for D-phenylalanine.

### 2.4. Crystallographic data of compound 1

Yellow crystals of **1** was obtained from MeOH. X-ray diffraction analysis was carried out on an Xcalibur Atlas Gemini Ultra diffractometer (Agilent Technologies) with Cu Kα radiation (λ = 1.54184 Å) at 293 (2) K. Crystal data for compound **1**: C_42_H_44_N_6_O_6_, *M* = 728.83; needle crystal (0.48 × 0.29 × 0.18 mm^3^), orthorhombic system, space group P 2(1), *a* = 9.3167 (2) Å, *b* = 19.4360 (3) Å, *c* = 21.0342 (3) Å, α = 90°, β = 90°, γ = 90°, *V* = 3808.86 (11) Å^3^, *Z* = 4, *d* = 1.271 g/cm^3^, *F*(000) = 1544. A total of 6701 reflections were collected in the range 6.2 ≤ 2θ ≤ 134.0, of which 5935 unique reflections with *I* > 2*σ(I)* were collected for the analysis. The structure was refined by full-matrix least squares on *F* ^2^ using SHELXL-2015 package software. The final reliability factors are: *R* = 0.0526, *wR_2_ *= 0.1451, Flack parameter = 0.07 (16), and the goodness of fit on *F*^2^ was equal to 1.037. Crystallographic data for the structure reported in this paper have been deposited at the Cambridge Crystallographic Data Centre under the reference number CCDC 1819264. Copies of the data can be obtained, free of charge, on application to CCDC, 12 Union Road, Cambridge CB2 1EZ, UK (fax: +44-(0)1223–336033 or email: deposit@ccdc.cam.ac.Uk).

### 2.5. ECD calculation

The CIF structure of compound **1**was initially optimised at B3LYP/6-31 + g (d, p) level in MeOH using the CPCM polarisable conductor calculation model. The theoretical calculation of ECD was conducted in MeOH using Time-dependent Density functional theory (TD-DFT) at the B3LYP/6-31 + g (d, p) level for one conformer of compound **1**. Rotatory strengths for a total of 30 excited states were calculated. ECD spectrum were generated using the program SpecDis 1.6 (University of Würzburg, Würzburg, Germany) and GraphPad Prism 5 (University of California San Diego, USA) from dipole-length rotational strengths by applying Gaussian band shapes with sigma = 0.3 eV.

### 2.6. Cytotoxicity bioassays

The cytotoxicity was measured by the MTT assay against the prostate cancer PC3 cell line. Tumour cell lines were seeded in 96-well plates (4 × 10^3^ per well in 100 μL). After 24 h of incubation cells were treated with gradient concentrations (100 μM, 50 μM, 25 μM, 12.5 μM, 6.25 μM, 3.125 μM) for another 72 h. Afterwards, MTT solution (5.0 mg/mL in RPMI-1640 media, Sigma, St. Louis, MO, USA) was added (20 μL/well) and then plates were incubated for another 4 h at 37°C. The compounds were dissolved in DMSO and cell growth inhibition assay was performed as reported previously. The growth inhibitory ability of the compounds was calculated and expressed using the IC_50_ value by the software of Dose-Effect Analysis. Doxorubicin (ADR) was used as a positive control (Wang et al. 2006).

## 3. Results and discussion

**Figure 2.**
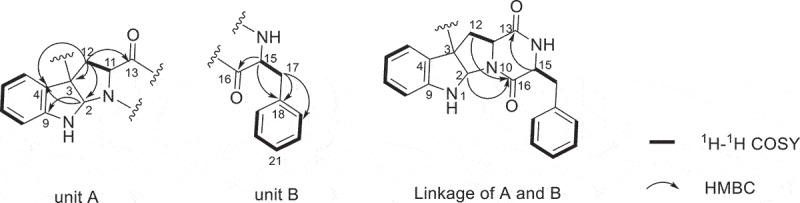
Partial structures of compound **1** based on ^1^H-^1^H COSY and HMBC spectra.

Compound **1** was purified as white powder. The ^1^H NMR and ^13^C NMR spectra showed 16 protons and 20 carbon signals, respectively (Tables 1 and 2, Figure S3 and S4, Supplementary data). While the quasi-molecular ion peak at 665.2868 for [M + H]^+^ obtained from ESI-HRMS revealed a molecular formula of C_40_H_36_N_6_O_4_ (calcd.665.2871 for C_40_H_37_N_6_O_4_), suggesting **1** to be a symmetric dimer. ^1^H-^1^H COSY cross peaks for H-5 (*δ*_H_ 6.94), H-6 (*δ*_H_ 6.72), H-7 (*δ*_H_ 7.19) and H-8 (*δ*_H_ 6.71), together with HMBC correlations from H-7 and H-8 to C-9 (*δ*_C_ 150.1), H-5 and H-6 to C-4 (*δ*_C_ 125.7) concluded one ortho-disubstituted benzene fragment. The tryptophan-like moiety (Unit A) was established by ^1^H-^1^H COSY cross peaks for H-12 (*δ*_H_ 2.17, 2.29) and H-11 (*δ*_H_ 1.89), combined with the key HMBC correlations of H-2 (*δ*_H_ 5.03) with C-3 (*δ*_C_ 58.0) and C-9, H-5 with C-3 and C-4, H-11 with C-3 and C-16 (*δ*_C_ 166.1), H-12 with C-13 (*δ*_C_ 169.5), C-2 (*δ*_C_ 78.2), C-3 and C-4 (Figure 2). The left signals of six olefinic carbon at *δ*_C_ 133.8 (C-18), 129.6 (C-19/C-23), 128.3 (C-20/C-22) and 126.8 (C-21), combined with the five olefinic protons at *δ*_H_ 6.98 (H-19/H-23), 6.85 (H-20/H-22), 6.84 (H-21) lead to a monosubstituted benzene fragment, which was connected to be a phenylalanine residue (Unit B) by the ^1^H-^1^H COSY cross peaks for H-15 (*δ*_H_ 4.13) and H-17 (*δ*_H_ 3.16, 2.83), together with HMBC correlations from H-15 to C-16 and C-18, H-17 to C-16, C-18 and C-19 (Figure 2). The HMBC correlations of H-15 with C-13 and H-2, H-12 with C-16 connected unit A with B, possessing an indole diketopiperazine monomer (Figure 2). The only left C-3 site was the position that connected the two monomers. Therefore, the planner structure of **1** was finally determined to be a twofold symmetrical dimer (Figure 1), which was the same as the known compound **4** (WIN 64821) (Barrow et al. 1993).

**Figure 3.**
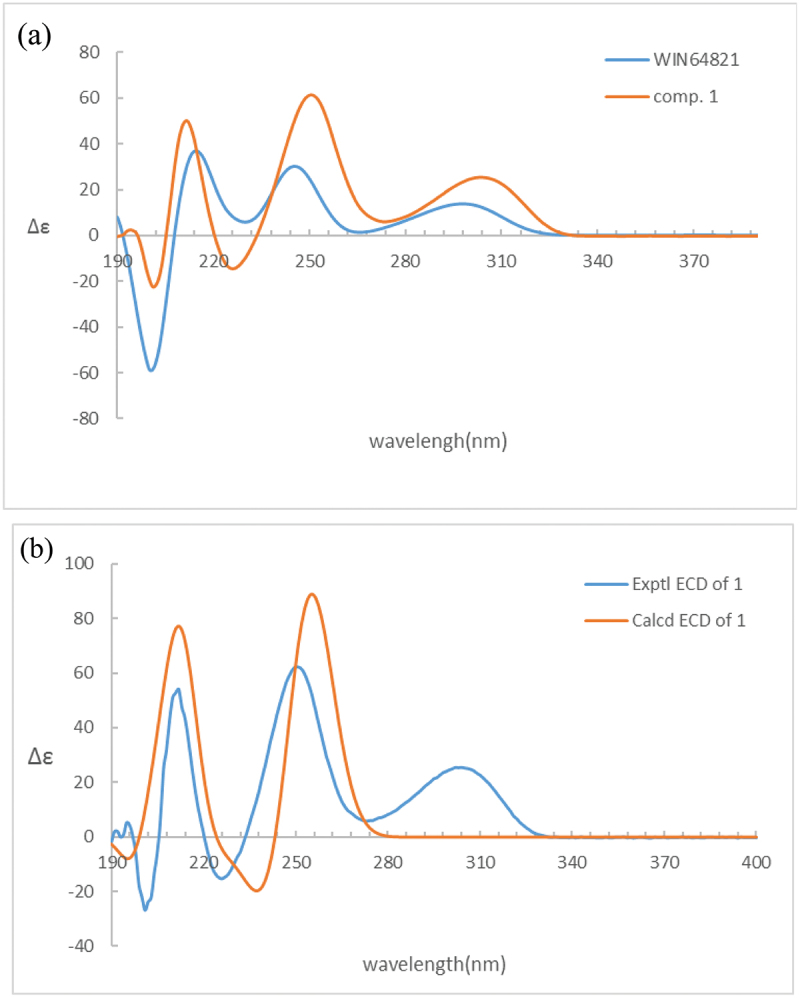
(a) ECD spectra of compounds **1** and **4**. (b) Experimental and calculated ECD spectra of compound **1.**

**Figure 4.**
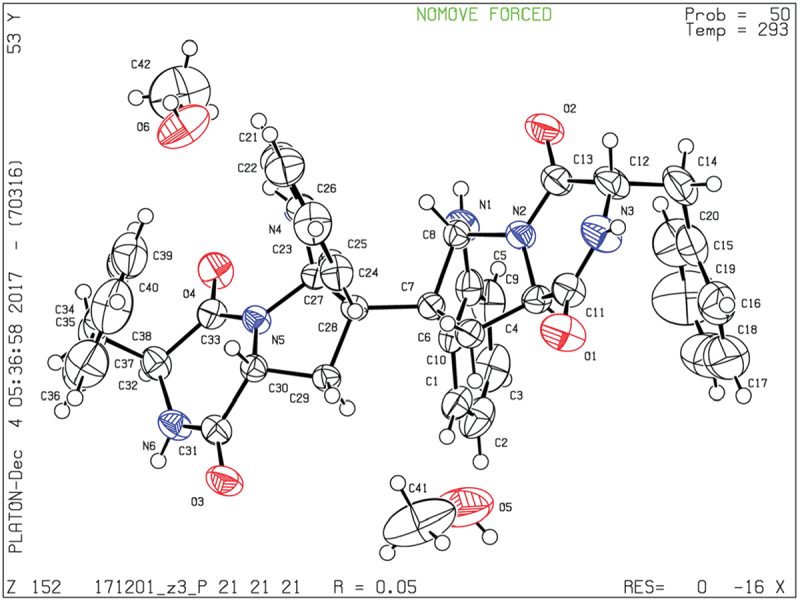
ORETP drawing of compound **1.**

Comparison of the ^1^H-NMR data between compounds **1** and **4** revealed a significant difference that there was an unusual up-field proton signal for H-11 (*δ*_H_ 1.89) in compound **1**. It was possibly caused by the positive shielding effect of the adjacent aromatic ring as the α-H of amino acid residue found in asperazine (Varoglu et al. 1997). It was reported that the positive or negative cotton effect at 254 nm and 300 nm in the electronic circular dichroism (ECD) spectrum depended on the stereochemistry of C-2/C-2’ and C-3/C-3’ (Barrow et al. 1993). Compound **1** showed positive cotton effect at 254 nm and 300 nm, same as WIN 64821, which indicated the chirality of C-2/C-2’ and C-3/C-3’ has not changed, both were *R* configuration (Figure 3a). The calculated ECD spectrum of this configuration at 254 nm was the same as experiment result, while the theoretical data at 300 nm did not yield (Figure 3b). It is speculated that the n-π transition signal of carbonyl is about 300 nm. The theoretical calculations suggested the carbonyl group had little effect on the configuration change may be the reason that no calculation results were obtained at 300 nm. The acid hydrolysates of compound **1** reacted with Marfey’s reagent (FDAA) showed the presence of L-phenylalanine residue by HPLC analysis (Figure S10, Supplementary data). Therefore, the absolute configuration at C-15/C-15’ was determined as *S*. The up-field shifting was speculated to be caused by the positive shielding effect of the aromatic ring of phenylalanine residue, so that H-11 and H-15 were in the opposite side of the piperazine ring. Because there was no change of chirality occurred at C-15/C-15’, the configuration at C-11/C-11’ were inverse to *R*, which was different from the reported compound WIN 64821. Finally, the speculation described above was proved by the X-ray crystallographic data (Figure 4) and the absolute stereochemistry of compound **1**, the symmetrical dimer, was determined as 2 *R*, 2ʹ*R*, 3 *R*, 3ʹ*R*, 11 *R*, 11ʹ*R*, 15*S*, 15ʹ*S*.

**Figure 5.**
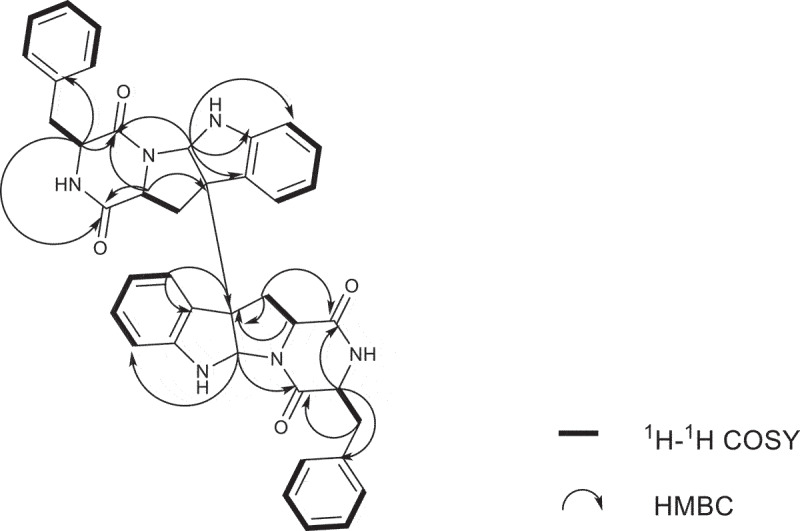
^1^H-^1^H COSY and key HMBC correlations of compound **2.**

Compound **2** was isolated as white powder. The quasi-molecular ion peak at 665.2863 for [M + H]^+^ obtained from ESI-HRMS revealed a same molecular formula of C_40_H_36_N_6_O_4_ (calcd. 665.2871 for C_40_H_37_N_6_O_4_) as compound **1**. The ^13^C NMR spectrum showed 40 carbon signals, combined analysis with DEPT and HSQC spectra revealed the presence of four amide carbonyls (*δ*_C_ 167.2, 168.9, 168.7, 165.7), four methylene carbons (*δ*_C_ 35.4, 36.7, 40.2, 40.4), six aliphatic methine carbons (*δ*_C_ 54.8, 56.6, 58.7, 58.3, 78.3, 80.3), two aliphatic quaternary carbons (*δ*_C_ 58.1, 58.5), six olefinic quaternary carbons (*δ*_C_ 126.4, 129.7, 148.4, 150.1, 134.5, 134.0) and eighteen olefinic methine carbons (Tables 1 and 2). It was found that the ^1^H and ^13^C NMR signals were twice that of compounds **1** and **4**, suggesting **2** to be an asymmetric dimer composed of two stereochemistry different monomers. The planner structure of compound **2** was elucidated by 1D and 2D NMR spectra (Figure 5). The ^1^H-^1^H COSY cross peaks for H-12 (*δ*_H_ 2.60, 2.24) and H-11 (*δ*_H_ 2.31), combined with the key HMBC correlations of H-2 (*δ*_H_ 4.83) with C-3 (*δ*_C_ 58.1) and C-9 (*δ*_C_ 148.4), H-5 (*δ*_H_ 7.08) with C-3 and C-4 (*δ*_C_ 129.7), H-11 with C-2 (*δ*_C_ 78.3), C-3 and C-13 (*δ*_C_ 168.7), H-12 with C-13 (*δ*_C_ 168.9), C-2 (*δ*_C_ 80.3), C-3 and C-4 constructed the tryptophan-like moiety (Figure 5). The ^1^H-^1^H COSY cross peaks for H-15 (*δ*_H_ 4.10) and H-17 (*δ*_H_ 3.19, 2.83), together with HMBC correlations from H-15 to C-16 (*δ*_C_ 167.2) and C-18 (*δ*_C_ 134.5), H-17 to C-16, C-18 and C-19 (*δ*_C_ 129.1) lead to a phenylalanine residue (Figure 5). The HMBC correlations of H-15 with C-13 and H-2, H-12 with C-16 connected tryptophan-like moiety with phenylalanine residue, possessing an indole diketopiperazine monomer (Figure 5). The left signals were used to elucidate the other monomer. Then the left C-3 site was the position that connected the two monomers. Therefore, the planner structure of **2** was finally determined, which was the same as compound **1** mentioned above.

**Figure 6.**
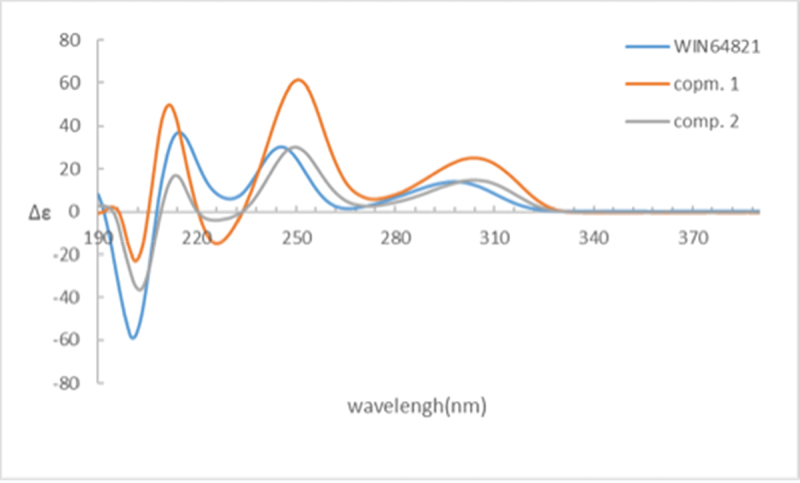
ECD spectra of compounds **1, 2** and **4.**

Detailed analysis of the ^1^H NMR spectrum of compound **2** revealed two unusual up-field proton signals for H-11 (*δ*_H_ 2.31) and H-11’ (*δ*_H_ 2.21), which were estimated to be affected by the positive shielding effect of the phenyl ring of phenylalanine residue as well according to the configuration of compound **1**. Therefore, H-11 and H-15, H-11’ and H-15’ were on the opposite side of the respective piperazine ring. Hydrolysis in HCl, followed by Marfey’s derivatisation, showed the presence of D- and L-phenylalanine residues in the structure of **2** (Figure S19, Supplementary data), which indicated the absolute configurations at C-15 and C-15’ were different. The ECD spectrum of compound **2** showed positive cotton effect at 254 nm and 300 nm (Figure 6), suggesting the chirality of C-2/C-2’ and C-3/C-3’ has not changed, still were *R*. Then the NOESY cross peak of H-2 (*δ*_H_ 4.83) and H-11 suggested the cis-orientation of the two protons, which further defined the absolute configuration at C-11 as *S*. For H-11 and H-15 were *trans*-orientation, the chirality of C-15 was determined as *R*. Simultaneously, C-15’ had the *S* configuration followed the results of Marfey’s method and the stereochemistry of C-11’ was deduced as *R*. Finally, the absolute configuration of compound **2** was defined as 2 *R*, 2ʹ*R*, 3 *R*, 3ʹ*R*, 11*S*, 11ʹ*R*, 15 *R*, 15ʹ*S* (Figure 1).

Compound **3**, sharing the same planner structure of the afore-mentioned compounds, was an asymmetric dimer (Figure 1). ^1^H NMR spectrum revealed one unusual up-field proton signals for H-11 (*δ*_H_ 1.98) and one normal proton for H-11’ (*δ*_H_ 4.02) (Table S1, Supplementary data). The up-field shifting was estimated to be caused by the same reason as that for compounds **1** and **2**, the positive shielding effect of the phenyl ring. The absolute configurations at C-15/C-15’ were both defined as *S* depending on Marfey’s results (Figure S29, Supplementary data). Thus, the chirality of C-11 and C-11’ was determined as *R* and *S*, respectively. The ECD spectrum of compound **3** showed positive cotton effect at 254 nm and 300 nm (Figure S30, Supplementary data), suggesting the chirality of C-2/C-2’ and C-3/C-3’ has not changed, still were *R*. Therefore, the absolute configuration of compound **3**, which was consistent with the compound reported in the literature (Gu et al. 2019), was defined as 2 *R*, 2ʹ*R*, 3 *R*, 3ʹ*R*, 11 *R*, 11ʹ*S*, 15*S*, 15ʹ*S*.

All the four compounds were tested for their in vitro cytotoxicity against the prostate cancer PC3 cell line by MTT method. Unfortunately, all of them were found to be inactive, displaying IC_50_ values >40 μM. Complex structural and stereoisomeric modification might influence the biological effects of the target compounds through the alteration of the shape or rigidity of the scaffold (Giessen et al. 2013; Giessen and Marahiel 2015). As some members of dimeric diketopiperazine encompass a vast spectrum of biological properties, it is suggested that the four compounds’ probability possesses an alternative and undefined biology that can be studied in further research.

## 4. Conclusion

Diketopiperazine scaffold, the smallest of all cyclopeptides, frequently embedded in complex metabolic architectures. Dimeric diketopiperazines are a relevant variation of this class of natural products occupying an enormous chemical complexity. So far, the biosynthesis of mainframe structure of diketopiperazine was found to be catalysed by nonribosomal peptide synthetases (NRPSs) and cyclodipeptide synthases (CDPSs) (Gondry et al. 2009; Belin et al. 2012; Borgman et al. 2019). Tailoring enzymes, including oxidoreductases and prenyltransferases, modify the initially assembled diketopiperazine, and cytochrome P450 responsible for the dimerisation of diketopiperazine monomers (Mishra et al. 2017; Shende et al. 2020). Compounds **1–4** were dimeric diketopiperazine stereoisomers, whose configuration was mainly different at C-11/C-11’ and C-15/C-15’ positions, while C-2/C-2’ and C-3/C-3’ positions were identical, indicating the biosynthesis of the four stereoisomers might be carried out by using different chiral phenylalanine and tryptophan as substrates, then dimerised by a possible P450 enzyme at C-3/C-3’ spot. Although the four dimeric diketopiperazine stereoisomers showed noncytotoxic against prostate cancer PC3 cell line, due to the diverse biological activities this class of compounds possess, they still provide attractive new targets for developing on the diketopiperazine scaffold to support future drug discovery.
